# Supplementary material for: Investigation to mitigate system-level factors contributing to hospital-acquired infection cases in the low-resource setting: A qualitative exploratory study in Bangladesh
Source: PLoS One. 2026 Apr 1;21(4):e0346013. doi: 10.1371/journal.pone.0346013 (PMC13042738; doi:10.1371/journal.pone.0346013)
Supplement: S2 File — (DOCX) [file pone.0346013.s002.docx]

**Informed Consent Form (KII)**

**Investigation to Mitigate System-Level Factors Contributing to Hospital-Acquired Infection Cases in the Low-Resource Setting: A Qualitative Exploratory Study in Bangladesh**

The study is being conducted under the direct supervision of Palash Chandra Banik, Associate Professor, Department of Noncommunicable Diseases, Bangladesh University of Health Sciences (BUHS), by Afsana Sultana and Fatema Tuz Johora, Department of Microbiology, BUHS. You have been selected for our Key Informant Interview in the qualitative phase of our study, for your role and experience in the hospital’s infection control and prevention management. During the study, you will be able to ask questions and raise your concerns regarding the study and your participation.

**Purpose:** The study aims to assess the knowledge, attitude, and practice (KAP) regarding hospital-acquired infection among nurses at a tertiary care hospital in Dhaka city.

**Risks and discomforts:** Participating in this study will not put you at any physical or psychological risk. All questions will be related to (1) demographic data, such as age, gender, and (2) your insight on hospital-acquired infection control measures.

**Benefits:** You will not get any direct benefit from this study. Nevertheless, the patients and society as a whole may benefit from the results of this study. The findings of this study may help to formulate policies to take preventive measures against hospital-acquired infections.

**Payment for participation:** No financial benefits will be received by participating in this study. But the results achieved will be used for your benefit in the future.

**Confidentiality:** All information regarding your research will be kept strictly confidential by us. In addition, only researchers and their collaborators will have access to this research data. If you wish to maintain anonymity, confidentiality will be maintained throughout the process. The data obtained from the interviews will be used to achieve the research objectives. These may be used in various publications, presentations, or government planning documents, but none of your personal information will be disclosed to anyone.

**Right of participation and exclusion:** Your participation in this study is entirely voluntary. You can remove yourself from the study at any time. You can also withdraw yourself during the question and answer session i.e. in between. Or you can skip certain questions if you don't want to answer them.

**Part of Consent**

1. I have read/listened carefully to the information provided by the researcher
2. I understand the research procedure and possible risk/discomfort related to the research
3. I agree to participate in this study and consent to audio tape or record my interview.

**Participant’s name:……………………………………**

**Participant’s mobile number:………………………………….**

**Signature……………………………………Date……………………**

**Researcher’s name………………………………….…………………….................**

**Researcher’s signature………………………………….. Date…………………….**

**In case of contact:** Please contact us if you have any questions or concerns related to the study.

Afsana Sultana: +8801756585082

Fatema Tuz Johora: +880 1994-239020

**Informed Consent Form (FGD)**

**Investigation to Mitigate System-Level Factors Contributing to Hospital-Acquired Infection Cases in the Low-Resource Setting: A Qualitative Exploratory Study in Bangladesh**

The study is being conducted under [direct supervision of Palash Chandra Banik, Associate Professor, Department of Noncommunicable Diseases, Bangladesh University of Health Sciences (BUHS), by Afsana Sultana and Fatema Tuz Johora, Department of Microbiology, BUHS. You have been selected for our focus group discussion in the qualitative phase of our study, for your role and experience in the hospital’s infection control and prevention management. During the study, you will be able to ask questions and raise your concerns regarding the study and your participation.

**Purpose:** The study aims to assess the knowledge, attitude, and practice (KAP) regarding hospital-acquired infection among nurses at a tertiary care hospital in Dhaka city.

**Risks and discomforts:** Participating in this study will not put you at any physical or psychological risk. All questions will be related to (1) demographic data, such as age, gender, and (2) your insight on hospital-acquired infection control measures.

**Benefits:** You will not get any direct benefit from this study. Nevertheless, the patients and society as a whole may benefit from the results of this study. The findings of this study may help to formulate policies to take preventive measures against hospital-acquired infections.

**Payment for participation:** No financial benefits will be received by participating in this study. But the results achieved will be used for your benefit in the future.

**Confidentiality:** All information regarding your research will be kept strictly confidential by us. In addition, only researchers and their collaborators will have access to this research data. If you wish to maintain anonymity, confidentiality will be maintained throughout the process. The data obtained from the interviews will be used to achieve the research objectives. These may be used in various publications, presentations, or government planning documents, but none of your personal information will be disclosed to anyone.

**Right of participation and exclusion:** Your participation in this study is entirely voluntary. You can remove yourself from the study at any time. You can also withdraw yourself during the question and answer session i.e. in between. Or you can skip certain questions if you don't want to answer them.

**Part of Consent**

1. I have read/listened carefully to the information provided by the researcher
2. I understand the research procedure and possible risk/discomfort related to the research
3. I agree to participate in this study and consent to audio tape or record my interview.

**Participant’s name:……………………………………**

**Participant’s mobile number:………………………………….**

**Signature……………………………………Date……………………**

**Researcher’s name………………………………….…………………….................**

**Researcher’s signature………………………………….. Date…………………….**

**In case of contact:** Please contact us if you have any questions or concerns related to the study.

Afsana Sultana: +8801756585082

Fatema Tuz Johora: +880 1994-239020

**Guideline for the qualitative phase**

1. What is the prevalence of hospital-acquired infections in your hospitals?
2. What are the most common types of HAIs observed in your hospital?
3. What is the current protocol for preventing hospital-acquired infection in your hospital?
4. What are the main challenges your hospital faces in preventing HAIs?
5. What are your goals for HAI prevention in the next 5 years?
6. What support or resources would be most beneficial in achieving your HAI prevention goals?
7. Is there anything else you would like to add about your experiences or insights into HAI

**Quotes from the interview supporting the identified themes**

| **Themes** | **Quotes** |
| --- | --- |
| Need for Systematic HAI Monitoring | *“We should first conduct surveys on this. In our country, surveys about basic things are not conducted. Honestly, it would be hard to say accurately without any survey, but consider that in this hospital, there is a rate of nosocomial infection or HAIs”*. (KII2)  *“Once we made an attempt in neurosurgery on how to decrease the rate of infection there. There was a time when neurosurgery meant that if an operation was conducted, 50% of people would have an infection; that’s why we attempted to decrease the infection. In the neuroscience department, the infection rate went from 15% to 10%. In surgery, it went from 20% to 5%. However, we did not conduct a survey overall but on a random basis in some wards. So, it would be difficult for me to tell you the whole scenario for hospital-acquired infection”*. (KII2)  *“We don’t have any statistical data on what percentage of patients are affected in this hospital.”* (KII1)  *“It is present, it is not 0%”. Added that, “To some extent, the infection rate exists*” (KII5)  *“We get so many infected patients that there’s no way to determine whether the infection was acquired outside the hospital or within. If I try to give a specific figure, it might be inaccurate”*. (KII3) |
| Overburdened Healthcare Workforce | *“We face significant manpower shortages. Since 1972, no additional staff have been appointed. Many positions have not been replaced after retirement, which has created a crisis. The government is taking steps to address this issue.”* (KII9)  “*We cannot handle all patient responsibilities, including personal hygiene and feeding. Patients should have a dedicated caretaker who ensures cleanliness and hygiene. Many don’t bathe or change clothes, which increases infection risks. We try to provide counselling and check if they have eaten properly, but there’s only so much we can do.*” (Nurse no 5, FGD) |
| Gaps in IPC Knowledge and Practice | *“When we ask the nursing supervisor, they say the training has been discontinued due to lack of funding.”* (FGD3)  “*We have management and people on waiting and everything. But as I mentioned, it’s the same situation. When they sit, they are suddenly very active, and when they don’t, we just close our eyes (ignore it).*” (KII3)  “*We mostly get our training from IGM; the government provides this. It has been a long time since the last training session was held. The last one was done in 2005 which I attendant but I do not know if there was any after that*” (KII4)  *“No measures are taken. It’s self-defence.”*  And added, *“Occasionally, but only a little. Everything can’t be taught at once.”* (KII3)  *“Meetings and reviews are conducted, though not monthly.”* (KII9)  *“Training is provided, but there’s no one to monitor the implementation of IPC practices. Monitoring responsibilities need to be assigned, but there’s no designated person for this role”.* (KII10) |
| Impact of Hospital infrastructure and overload | *“The hospital is a 130-year-old building, and this is not the hospital structure; this was a governing body’s office of Assam state. During the 2nd World War, it was converted into a 200-bed hospital. But it's not a hospital structure; the height and ventilation of the building were not for a hospital. The architecture of hospitals is different since the amount of sunlight, air and lighting in the hospital is specified”* (KII2)  *“As numerous people are constantly moving here, including staff, workers, outsiders, etc., some degree of infection rate is inevitable.”* (KII5)  *“If the environment were clean, then we would not have germs here. In the hospital, during the initial period when there were operations, there was no need for antibiotics. But now, there is no hospital in Bangladesh that does not inject 2gm injection before (an operation)”.* (KII2)  “It is already difficult. Suppose if you go to the pediatric ward, you will see there are 3 patients in 1 bed, in the obstructive ward, 2 patients in 1 bed and in the surgical ward, 2 patients in 1 bed so, this does not fall in any standard procedure and becomes very difficult”. (KII1)  *“There are too many visitors. For 1 patient, there are 4/5 visitors”,* and sharing her experience, she stated, *“One night while I was doing my duty, a stand almost fell on my head due to too many visitors.”* (Nurse no 1, FGD1) |
| Hospital Hygiene | *“Regarding hospital waste management, we have several colour-coded bins that indicate where to dispose of what type of waste. However, we are not provided with any special training on waste management.”* (KII10)  “Yes, we do. But for patients and their relatives, we can't maintain them that much”. (Nurse no. 2, FGD)  *“Here the people who come with a cough, just so they don’t cough their sputum anywhere, we have place bin in different places so that they can cough their sputum there”.* (KII7) |
| Consumables and Resource constraint | “*No, sufficient protection measures are not provided*”. And added that, “*We lack a lot of supplies. This creates many necessities*” (KII3)  “For me to provide proper care, I need to be taken care of properly, provided with the proper materials” (Nurse 1, FGD)  “*The government has improved some aspects. For example, many medications are now provided free or at very low costs. However, supplies like gloves, cleaning materials, and even proper ventilation are still lacking. We try to keep the hospital as clean as possible, but with limited resources, it’s difficult”*. (Nurse no5, FGD)  “*The amount I have, as I said, of course, we might not be able to provide fully but never would have happened where we couldn’t provide Savlon (rubbing alcohol)”*(KII2)  *“We cannot provide many safety measures, as we have to take care of 3 patients instead of 1. And we do not know who does and who doesn’t have the infection, so there is a continuous risk”*.  “*Nowadays, we are provided with protective equipment. There was a time when there was no supply of gloves. We were also cautious and worried about our safety. After COVID-19, many people were more conscious. Now, PPE are provided, but gloves and masks are provided more*”. (KII4)  “*Some patients stay for 10 days, 15 days, or even a month, and the same catheter continues to be used throughout their stay. This significantly increases the risk of catheter-related infections.*” (KII10) |
| Common HAIs and At-Risk Groups | *“Consequently, the infection rate is very high in post-operative surgical care, especially in the gyno department.”* (KII5)  *“For females, uterine infection. For smokers or males, Respiratory Tract Infection. We have also observed some in gyno that is post-operative infection”*. (KII6)  “*Children are most at risk. And mostly elderly people, people with diabetes and heart conditions are at risk*.” (KII8)  *“Some patients in the ICU do develop HAI occasionally. Compared to other wards, the number of HAI cases here is quite low.”* (KII9)  “*The ICU has critically ill patients, including those with tuberculosis and other severe diagnoses. Many of them don’t even know they are infected until we investigate. We sometimes find out that a patient is HBsAg positive (Hepatitis B positive) only after further tests, but we still have to care for them”*. (Nurse no 2, FGD) |
| Healthcare Worker Safety Concerns | “*The ICU has critically ill patients, including those with tuberculosis and other severe diagnoses. Many of them don’t even know they are infected until we investigate. We sometimes find out that a patient is HBsAg positive (Hepatitis B positive) only after further tests, but we still have to care for them”* (Nurse no 2, FGD)  *“It is difficult to pinpoint blame. The system itself has several flaws. The lack of equipment and visitor control all contribute to the problem. Everything is interconnected*”. (Nurse no 3, FGD)  “People spit everywhere”. (KII3)  *“One of several reasons is the failure to maintain a restricted area or sterility and infection-prevention control checking in the operating theatre due to various reasons.”* (KII5)  *“Most of the people who come to government hospitals for treatment are from middle, lower-middle, or even poorer socioeconomic backgrounds. They are generally not health conscious. Even when we instruct them, they often do not follow protocols.”*  The nurse informant added, “*We guide their (infectious patients) families to maintain distance and wear masks, but they rarely follow these guidelines, making them highly susceptible to infections.*” (KII10)  “*When a patient comes to a TB hospital, the patient knows that there are TB patients, so they wear masks out of self-awareness. And people who work here wear masks themselves. But in other hospitals, no one thinks about this risk; as a result, they do not take measurements. For this reason, it can be seen that other hospitals are not as conscious as our hospital*”. (KII7) |
| Factors influencing the rate of HAIs | *“During the daytime the population in Dhaka Medical is, can you imagine, 40,000 people come and go in this building Dhaka Medical. Each of my 4300 patients has at least 2/3 attendants, in outdoor 5000 patients come and each of them brings 2/3 attendees, and then indoors, a total of 30,000 here and my staff is 6000, answer, reporters, and hawkers, in total 40,000 people. Now that 40,000 people have walked here and spread dust, with my manpower cleaning this, how many teams are prepared?”,* (KII2)  *“Reducing visitor numbers is essential, but it’s difficult. Every patient’s family thinks they deserve special access.”* (FGD5)  *“We are not aware, as doctors, we have some shortcomings, careless sometimes. (for example) I came and said, “Let's see, open your bandage,” and then pulled it away. The way nurses should dress, if they do not do it properly or let a ward boy do it, many times you can see there is malpractice. The training of a nurse and a ward boy is not the same; a ward boy is not supposed to dress.”* (KII2)  *“Nurses do not follow the protocol, and neither do the fourth-grade employees. Even when we try to teach them individually, they do not comply.”* (KII10)  *“The people of Bangladesh do not like preventive health education. They come to the doctor to ask for medicine. If you tell them not to smoke or lose some weight, the patients wouldn’t like it; rather, the patient wants to have 2 vitamin capsules or an antibiotic. So this is the scenario.”* (KII2)  *“If one patient has a skin infection, the proximity between patients often allows the infection to spread easily. Even when we provide instructions, they often fail to maintain proper hygiene.”* (KII10) |
| Strategies for Reducing HAIs | *“For patients, if there is no awareness at the community level. If I teach one patient today, they will leave tomorrow, and another new patient will come; we need a system for health educators. There is no continuous health education.”* (KII2)  “This *is an integrated approach; you and we should adopt a holistic approach, and only then will the IPC be effective. The first is, as I said, a lack of grooming and a theoretical approach. WHO always considers leadership as one of the pillars in hospital management. We should make these strategies integrated in national policies and make the infection control strategies firm and monitor them to ensure they’re working”*. (KII2)  *“Around 50% of the patients admitted to Dhaka Medical are emergency patients. By communicating with the ministry through mail and discussion, we are trying to reduce the load of patients. Another way is to build a new hospital, which is already in progress and hopefully will be on for construction soon. It is a 5000-bed hospital*”. (KII1)  *“Administrative steps could help, such as counselling patients during admission and restricting one attendant per patient. Attendants could visit occasionally but not stay continuously”.* (KII3)  *“This awareness needs to start from home and needs to be created among children so that, at the end of the day, even a child knows not to touch a wound with dirty hands”*. (KII8)  “*Firstly, awareness needs to be increased. Second, facilities need to be increased. By facilities, I mean that OT and post-operative facilities need to be increased. Thirdly, all the instruments and equipment need to be autoclaved properly. Finally, operation-related infection mainly occurs in the hospital; all staff related to the operation should properly disinfect themselves before entering the OT.”* (KII6)  “*Dedicated IPC committees and a multidisciplinary approach are needed. Super-specialities, such as clinical microbiologists and infectious disease specialists, should be developed. IPC committees also need administrative power to implement changes effectively. Hospitals should follow IPC standards based on their settings and patient admission patterns. He proposed that “Regular training and awareness programs for doctors and nurses are also essential. For instance, complacency can lead to risky practices, such as drawing blood from HIV patients without gloves. These weak points need to be addressed through regular monitoring and corrective actions.”* (KII8)  *“To improve what we can do is, the video that we have here, the videos related to health education, the health education we are giving here, can be transformed into videos in different places to create awareness. In our wards, where the patients usually sit, we can create awareness through TV. We can do this for the patients. And regarding hospitals, the thing we can do is we can create awareness at the government level, then that would be more helpful, like we used to create awareness about diarrhoea through a government initiative, by showing it repeatedly on BTV (Bangladesh Television Channel). If we create awareness like this, then the patients will receive the correct information. Now people are very active on Facebook, so we can create awareness through Facebook”.* (KII7) |
