# Supplementary material for: Investigation to mitigate system-level factors contributing to hospital-acquired infection cases in the low-resource setting: A qualitative exploratory study in Bangladesh
Source: PLoS One. 2026 Apr 1;21(4):e0346013. doi: 10.1371/journal.pone.0346013 (PMC13042738; doi:10.1371/journal.pone.0346013)
Supplement: Attachment — Submitted filename: Response to reviewers.docx [file pone.0346013.s004.docx]

Investigation to Mitigate System-Level Factors Contributing to Hospital-Acquired Infection Cases in the Low-Resource Setting: A Qualitative Exploratory Study in Bangladesh

**Editor’s comments**

| Reviewer’s comments | Authors’ response |
| --- | --- |
| 1. Please ensure that your manuscript meets PLOS ONE's style requirements, including those for file naming. The PLOS ONE style templates can be found | Following PLOS ONE style requirements, the manuscript has been revised accordingly. The Abstract has been formatted under a single heading, headings and font sizes have been adjusted to match the journal template, and table legends have been added where appropriate.  The title/cover page has also been updated in line with PLOS ONE guidelines. A symbol legend for author contributions was not included, as no authors contributed equally, and all authors share the same institutional affiliation and address. |
| 2. We note that you have provided additional information within the Acknowledgements Section that is not currently declared in your Funding Statement. Please note that funding information should not appear in the Acknowledgments section or other areas of your manuscript. We will only publish funding information present in the Funding Statement section of the online submission form.   Please remove any funding-related text from the manuscript and let us know how you would like to update your Funding Statement. | As requested, we have removed all funding-related text from the Acknowledgements section. No funder name or reference number now appears in the manuscript outside the Funding Statement. (page #21).  The Funding Statement provided during online submission remains accurate and therefore requires no update. |
| 3. Please state what role the funders took in the study.  If the funders had no role, please state: "The funders had no role in study design, data collection and analysis, decision to publish, or preparation of the manuscript."   Please include this amended Role of Funder statement in your cover letter; we will change the online submission form on your behalf. | The Role of the Funder statement has been clarified in the Methods section as requested. The revised text now reads:  “The funders had no role in the study design, data collection and analysis, decision to publish, or preparation of the manuscript.”  (page #8). |
| 4. We note that you have indicated that there are restrictions to data sharing for this study. PLOS only allows data to be available upon request if there are legal or ethical restrictions on sharing data publicly.   a) If there are ethical or legal restrictions on sharing a de-identified data set, please explain them in detail (e.g., data contain potentially identifying or sensitive patient information, data are owned by a third-party organization, etc.) and who has imposed them (e.g., a Research Ethics Committee or Institutional Review Board, etc.). Please also provide contact information for a data access committee, ethics committee, or other institutional body to which data requests may be sent.  b) If there are no restrictions, please upload the minimal anonymized data set necessary to replicate your study findings to a stable, public repository and provide us with the relevant URLs, DOIs, or accession numbers. For a list of recommended repositories, please see | As stated in the informed consent form, strict confidentiality was assured to all participants. Identifiable information, including names, contact details, workplace identifiers, signed consent forms, and audio recordings, will be securely stored by the designated corresponding author and will not be shared publicly. Audio recordings and consent documents will be made available to the journal editorial office only upon reasonable request for verification purposes, in accordance with ethical approval and participant consent. Therefore, public sharing of audio files and consent documents is ethically restricted. These restrictions arise from the conditions set out in the participant-informed consent process, which specified that identifiable qualitative data would be accessed only by the research team and used solely for research purposes. These procedures were reviewed and approved by the Ethical Review Committee of the Bangladesh University of Health Sciences. Access to these data may be granted upon reasonable request to the corresponding author and the ethics committee, subject to approval by the participants.  To comply with PLOS ONE’s data-sharing requirements and to enable replication of the study’s findings, we will provide an excerpt from the qualitative codebook generated using Taguette, illustrating key codes, definitions, and anonymized examples, as supplementary materials. These files contain no personally identifiable information and are sufficient to reproduce the analytic process and results reported in the manuscript.  To ensure long-term and independent access to the minimal dataset in line with PLOS ONE’s Data Policy, requests for access to the restricted data can be directed to a non-author institutional point of contact- erc@buhs.ac.bd |
| 5. If the reviewer comments include a recommendation to cite specific previously published works, please review and evaluate these publications to determine whether they are relevant and should be cited. There is no requirement to cite these works unless the editor has indicated otherwise. | Thank you for this comment. Although the reviewers did not recommend citing specific previously published works, we reviewed the relevant literature and added additional references where appropriate to strengthen the manuscript. |

**Reviewer #1 comments**

| Reviewer’s comments | Authors’ response |
| --- | --- |
| 1. Study Design and Reporting (COREQ Adherence)  The paper presents an inductive grounded-theory approach and semi-structured guides, with purposive sampling across four tertiary sites. While this is appropriate and clearly articulated, core elements of qualitative reporting per the COREQ (Consolidated Criteria for Reporting Qualitative Research) checklist are currently underdeveloped. Examples include-  * Researcher characteristics and potential influence on data collection. * Nature of the relationship (if any) between interviewers and participants. * Setting of interviews (e.g., location, privacy conditions). * Whether the interview guide was pilot-tested or iteratively refined.  Since the adherence to COREQ is a standard practice for studies of this nature, I strongly recommend the authors to include a COREQ checklist as a supplementary file and revise the Methods section to align with its domains. | Thank you for this comment. We have now added the COREQ checklist as a supplementary file. |
| 2. Sample Size and Saturation  The study includes 10 key informant interviews (KIIs) and 1 focus group discussion (FGD) with 6 nurses. The authors state that data collection continued until saturation was reached. Please clarify how “theoretical saturation” was operationally defined (e.g., no new codes/themes emerging across the last few interviews). | Thank you for this comment. We have now clarified how theoretical saturation was operationally defined in the Methods section. Saturation was determined when no new codes or themes emerged during consecutive interviews. After the eighth key informant interview, no additional themes were identified, and the final interviews and the focus group discussion confirmed existing findings. Relevant text has been added to the manuscript (page #4). |
| 3. Data Collection Procedures  The manuscript reports face-to-face interviews, their durations, and describes consent procedures well. However, a few critical methodological details require clarification:  * Who conducted the interviews? What training did they have in qualitative methods? * Were the interviews held in private settings to mitigate social desirability bias? * Did the interview guides evolve as data collection progressed (i.e., iterative refinement)? | Thank you for this comment, we have followed the COREQ adherence and added the following lines.  *“The KIIs and FGD were conducted by the first two authors, both the female authors held bachelor's-level degrees and were employed as research assistants. The interviewers had prior exposure to qualitative research methods and underwent training, including a pilot (trial) interview with intern nurses, to refine the interview guide and ensure quality in data collection.” (Page #4)  *“Interviews took place in quiet private rooms (for KII) and a quiet area (for FGD) with the participant and research team” (Page#5)  *The semi-structured interview guide consisted of predefined core and flexibility was maintained through follow-up probes and clarifying questions based on participants’ responses. Minor refinements were made to probing and follow-up questions to enhance clarity. For instance, during our 6^th^ interview, when one participant mentioned reusing syringes, the question was later used as a probe under the core question “What are the main challenges your hospital faces in preventing HAIs?” |
| 4. Data Analysis and Tools  The use of constant comparative analysis is appropriate and well-described. However, there are a few inconsistencies and opportunities for improvement:  * The software cited, “Tageutte,” appears to be a misspelling. It is likely “Taguette.” Please confirm and correct this throughout the manuscript, and provide version details and URL (as is customary for citing statistical software) * Including an excerpt from the codebook (as supplementary material) would strengthen the transparency of the analytical process. | We apologize for the misspelling; the correct software name is indeed **Taguette**, and this has been corrected throughout the manuscript. A formal citation has also been added. Additionally, the codebook generated using Taguette will be provided as supplementary materials (page #6). |
| 5. Ethics and Consent  Explain why both written and verbal consent were obtained. For example, was verbal consent needed to confirm agreement to audio-recording? Also, indicate whether consent forms are available on request, in line with PLOS data availability policies. | Both written consent and verbal consent were obtained for audio recording. Written informed consent was obtained prior to participation and served as formal documentation of consent for the study. In addition, verbal consent was obtained immediately before audio recording to reconfirm participants’ willingness to be recorded. This step ensured voluntary and informed agreement at the time of recording.  As stated in the informed consent form, strict confidentiality was assured to all participants. Identifiable information, including names, contact details, workplace identifiers, signed consent forms, and audio recordings, will be securely stored by the designated corresponding author and will not be shared publicly. Audio recordings and consent documents will be made available to the journal editorial office only upon reasonable request for verification purposes, in accordance with ethical approval and participant consent. Therefore, public sharing of audio files and consent documents is ethically restricted. These restrictions arise from the conditions set out in the participant-informed consent process, which specified that identifiable qualitative data would be accessed only by the research team and used solely for research purposes. These procedures were reviewed and approved by the Ethical Review Committee of the Bangladesh University of Health Sciences. Access to these data may be granted upon reasonable request to the corresponding author and the ethics committee, subject to approval by the participants.  To comply with PLOS ONE’s data-sharing requirements and to enable replication of the study’s findings, we will provide an excerpt from the qualitative codebook generated using Taguette, illustrating key codes, definitions, and anonymized examples, as supplementary materials. These files contain no personally identifiable information and are sufficient to reproduce the analytic process and results reported in the manuscript.  To ensure long-term and independent access to the minimal dataset in line with PLOS ONE’s Data Policy, requests for access to the restricted data can be directed to a non-author institutional point of contact- erc@buhs.ac.bd |
| 6. Interpretation and Policy Implications  The manuscript notes that staff at the TB hospital “showed greater adherence to the IPC protocol,” attributing this to higher community awareness. However, it also states that “no studies have yet been conducted” to examine this link. This creates a tension between implied causality and admitted uncertainty. To maintain scientific clarity, this should be reframed as a hypothesis generated from qualitative insights, rather than an established effect. For example, the authors might say: “Participants perceived greater IPC adherence at the TB hospital, potentially linked to community awareness. This observation warrants further empirical evaluation to determine its validity and generalizability.” | Thank you for this important comment. We have revised the text to avoid implying causality and to clearly present this finding as a perception reported by the participants.  “Participants perceived that healthcare workers, patients, and visitors at the TB hospital demonstrated good adherence to IPC protocols, which they attributed to higher awareness of TB-related hazards in the community. However, as no empirical studies have yet examined the role of community awareness in influencing IPC adherence, this observation should be interpreted as a hypothesis generated from qualitative insights that warrants further investigation”, (page #19) |

**Reviewer #2 comments**

| Reviewer’s comments | Authors’ response |
| --- | --- |
| 1. The first sentence of the introduction is a strong opening, but could benefit from one or two relevant citations to back up the claim beyond general statements | Thank you for this comment. We have added 2 more references that the first sentence was based on (page #3). |
| 2. “Among the total postoperative patients, an alarming rate of 41.7% developed a surgical site infection [4].” ⟶ Please consider whether “alarming” is too strong for a scientific tone. A more neutral phrasing could be “a high rate of…” | As per the comment, we have changed “an alarming” to “a high”. (page #3) |
| 3. “This emphasises why preventing HAIs by maintaining IPC measures is crucial…” ⟶ This sentence would benefit from specifying how IPC contributes to economic and health system stability. | Thank you for this comment. We revised the sentence as follows: “This emphasises why preventing HAIs by maintaining IPC measures is crucial for the security of public health and the country's economy, as HAIs increase healthcare costs, and place additional strain on already limited health system resources.” (Page #3) |
| 4. “For this study, grounded theory with an inductive approach was adopted…”⟶ You may briefly clarify what grounded theory offers for this topic—i.e., why it is suitable for studying system-level factors. | To clarify what grounded theory offers for this topic we added the following sentence in methods “This approach was well-suited for examining system-level factors, as it focuses on understanding processes, interactions, and organizational dynamics and allows theory to emerge from healthcare service providers' experiences, particularly where established models do not fully capture contextual and institutional influences.” (Page #4) |
| 5. “Ethical permission was taken from the Ethical Review Committee, Bangladesh University of Health Sciences…” ⟶ Please mention the ethical approval number in parentheses. | As per the comment, we have revised to (BUHS/ERC/EA/24/60) (page #5) |
| 6. The constant comparative method was adopted…” ⟶ Please add a citation here for readers unfamiliar with the method. | As per the comment, a citation on the constant comparative method was added. (Page #6) |
| 7. “Two investigators independently reviewed the primary codes to form axial coding…” ⟶ Please add how disagreements were resolved (e.g., consensus, third reviewer). | As per the comment, the following line was added: “Disagreements that were not resolved through consensus between the two investigators were resolved by the third investigator” (page #6) |
| 8. The codes were used to identify patterns among them and generate categories and, eventually, themes.” ⟶ Please clarify the analytic flow (e.g., from open codes → axial categories → selective themes) for transparency. | Thank you for this suggestion. We have clarified the analytic flow from codes to categories and themes by adding an explanatory legend to the codes–categories–themes table, detailing the progression from open coding to category development and theme generation. (Page #7) |
| 9. “For data management and analysis, the software Tageutte was used.”⟶ Please check spelling: did you mean “Taguette”? If so, you might also cite the software formally. | We apologize for the misspelling; the correct software name is **Taguette**, and this has been corrected throughout the manuscript. A formal citation has also been added. In addition, the coded transcripts and the codebook generated using Taguette will be provided as supplementary materials (page #6). |
| 10. “In the KIIs, the minimum age and work experience in their role were 28 years and 7 months, respectively (Table 2).” – Please report a range or mean ± SD instead of only the minimum values. | As per the comment, was added, and the changes follows as, “the mean ± SD of age and work experience in their role were 49.6 ± 10.4 and 25.3 ± 11.8, respectively **(Table 2)**. In the FGD, the mean ± SD of age and experience of the nurses were 41.3 ± 10.1 and 17.3 ± 8.4” (page #8) |
| 11. If possible, please include a summary table of key themes, subthemes, and representative quotes to improve the readability. | Thank you for this suggestion. We have included a column for “quotes” in **Table 1**. (page #6,7) |
| 12. “Most participants admitted the presence of HAIs… but were unable to provide reliable or specific data.” – This is a critical insight. Consider linking this finding to the absence of a routine data audit. This helps frame it as a structural failure rather than an anecdotal gap. | Thank you for highlighting this point. We have revised the text into the following: “most participants admitted the presence of HAIs in their hospital but were unable to provide reliable or specific data, which they attributed to the absence of routine surveillance, standardized data audits, or regular prevalence studies at the institutional level”, (page #10) |
| 13. “The training isn’t regular and is usually reserved for nurses in senior positions.” – This quote is powerful and actionable. Please consider distinguishing between knowledge gaps due to poor cascade training vs. a complete lack of training infrastructure in the discussion. | As per the comment, the revised sentence is “While senior staff may receive IPC instruction, the absence of formal knowledge-transfer systems limits dissemination to junior and support staff who also play an important role in HAI prevention. Additionally, irregular training schedules point to the lack of a structured, system-wide IPC training programme”, (page #11) |
| 14. Theme 4 and theme 6 shows conceptual overlap. Please consider combining the themes into a larger theme. | We appreciate the reviewer’s observation regarding the potential conceptual overlap between Theme 4 and Theme 6. Our analytic approach aimed to identify and preserve specific and actionable constraints affecting IPC practices. Theme 4 captures structural and infrastructural limitations related to physical space and hospital design, whereas Theme 6 reflects material and operational resource shortages. Maintaining this distinction allows for more precise interpretation and policy-relevant recommendations.  To address the reviewer’s concern, we have revised the theme 6 title to “**Material and Resource Constraints**.” (Page #13) |
| 15. For the theme 10, recommendations, these are quite long and anecdotal. Please consider regrouping in policy-level, facility-level or community engagement strategies.. etc. | We thank the reviewer for this constructive suggestion. We have revised Theme 10 to reduce anecdotal descriptions and reorganised the findings into analytically distinct subthemes: policy-level, facility-level, and community engagement strategies in Table 1. (Page #7) |
| 16. “HAIs impact the health of both healthcare workers and patients…which is why the prevention of HAIs is key…”- This is kind of repetition of general knowledge. Please reframe it to avoid redundancy. | As per the comment, to avoid redundancy, the sentence has been removed. Currently, the sentence has been revised as follows: “The prevention of HAIs is key to improving the healthcare system of a country.”, (page #16) |
| 17. “According to this study’s findings, currently, there is no accurate database…”– This is important but needs clearer linkage to national policy implications. | As per the comment, the revised text is as follows,  “This reflects a wider national gap in infection surveillance, as other studies also suggest that about 90% of the tertiary care hospitals in Bangladesh do not strictly monitor the IPC measures or even have an audit system, and only 30% have an IPC training program regularly [12,13]. Strengthening national surveillance and mandating routine reporting of hospital-acquired infections should therefore be prioritized within Bangladesh’s health policy framework to improve patient safety and healthcare quality.”, (page # 17) |
| 18. “Besides uncheck HAI rate and gaps in IPC knowledge…”– Minor typo: should be “unchecked HAI rate.” Also, I suggest more formally framing this as “unmonitored HAI burden.” | Thank you for the comment. The text has been revised as “unmonitored HAI burden”, (page #17) |
| 19. The sentence “However, no studies have yet been conducted to understand the role and impact of community awareness in maintaining IPC measures…”– This is a strong knowledge gap; please highlight it earlier and frame it as a future research priority. Consider referencing any literature on community-based IPC if available. | Due to concerns raised regarding the creation of tension between implied causality and admitted uncertainty. To maintain scientific clarity, this was reframed as a hypothesis generated from qualitative insights, rather than an established effect. We have revised this part as follows: “Participants perceived that healthcare workers, patients, and visitors at the TB hospital demonstrated good adherence to IPC protocols, which they attributed to higher awareness of TB-related hazards in the community. However, as no empirical studies have yet examined the role of community awareness in influencing IPC adherence, this observation should be interpreted as a hypothesis generated from qualitative insights that warrants further investigation”, (page #19) |
| 20. The section about social obligations and visitors – This is contextually rich. However, consider distinguishing between cultural determinants and structural health system failures; both deserve separate policy solutions. | Thank you for these valuable suggestions. We have revised the section to clearly distinguish between cultural determinants (e.g., community engagement and visitor behavior) and structural health system limitations, emphasizing that each requires distinct policy responses (page #19) |
| 21. Triage and zoning – Excellent recommendations. However, I suggest aligning them with WHO IPC guidance or WASH in health care facilities guidelines. | We have aligned recommendations on triage and zoning with WHO IPC and WASH in health care facilities guidance to strengthen their evidence base. “In line with the WHO infection prevention and control (IPC) and WASH in health care facilities guidelines, the zoning method should be adopted”, “Furthermore, implementing the structural triage recommended by WHO for low-resource settings will also assist in addressing manpower and medical equipment shortages”, (page #19) |
