# Supplementary material for: Investigation to mitigate system-level factors contributing to hospital-acquired infection cases in the low-resource setting: A qualitative exploratory study in Bangladesh
Source: PLoS One. 2026 Apr 1;21(4):e0346013. doi: 10.1371/journal.pone.0346013 (PMC13042738; doi:10.1371/journal.pone.0346013)
Supplement: Attachment — Submitted filename: Response to Reviewers- Round 2.docx [file pone.0346013.s005.docx]

Investigation to Mitigate System-Level Factors Contributing to Hospital-Acquired Infection Cases in the Low-Resource Setting: A Qualitative Exploratory Study in Bangladesh

**Reviewer #2 comment**

| Reviewer’s comments | Authors’ response |
| --- | --- |
| 1. I appreciate the authors’ thoughtful revision in response to my comments. The expanded justification for the use of grounded theory and the added emphasis on system-level processes, interactions, and organizational dynamics strengthen the rationale and demonstrate careful consideration of the earlier comment.  However, the justification remains somewhat abstract and would benefit from greater methodological specificity. In particular, it would be helpful for the authors to clarify how grounded theory was operationalized in this study and what it uniquely enabled beyond a descriptive qualitative approach (e.g., thematic analysis) in this study. | Thank you for this valuable suggestion. We have revised the method section to clarify how grounded theory was operationalized (Page 4, lines 77-80) and the discussion section to clarify how this approach enabled the development of a contextual explanation of system-level drivers of hospital-acquired infections beyond a descriptive thematic account. (Page 19, lines 443-448) |
